# Hydatidosis: A Rare Case of Multi-organ Involvement

**DOI:** 10.7759/cureus.57562

**Published:** 2024-04-03

**Authors:** Harshitha Reddy, Suprit Malali, Rushikesh H Dhondge, Sunil Kumar, Sourya Acharya

**Affiliations:** 1 Internal Medicine, Jawaharlal Nehru Medical College, Wardha, IND

**Keywords:** management, diagnosis, cystic lesion, echinococcus granulosus, peritoneal hydatid cyst

## Abstract

Echinococcus granulosus is the tapeworm that causes hydatidosis. The liver is the most frequently impacted region, although it can also affect the spleen, lung, and peritoneum. Dogs are the definite hosts, whereas humans are the unintentional accidental hosts. The peritoneum is an unusual site for hydatid cysts. We report the case of a 42-year-old male who had abdominal distension. A CT scan revealed hydatid cysts in the liver, spleen, and peritoneum. The patient was managed conservatively with albendazole and advised for surgical intervention and removal of daughter cysts. This case highlights the uncommon presentation of hydatid disease involving multiple intra-abdominal organs concurrently. The successful management of such cases necessitates a multidisciplinary approach, encompassing accurate diagnosis, timely intervention, and comprehensive treatment strategies. Furthermore, this case emphasizes the importance of clinical suspicion in endemic regions to optimize patient outcomes and enhance quality of life.

## Introduction

The main cestode worm responsible for hydatid disease is Echinococcus granulosus, a dog tapeworm. Four species of Echinococcus cause infections in humans; the most prevalent species, E. granulosus and Echinococcus alveolaris, cause cystic and alveolar echinococcosis [1]. Cystic echinococcosis can infect any organ. Although it can infect any organ, the liver and the lungs are the primary sites where larvae settle. The larvae that pass through the intestinal wall through the portal flow are retained in the liver, and the structure of the cyst is formed. It has been observed that larvae can enter the abdominal cavity by invading the intestinal walls. After that, they may move via the lymphatic system and settle in various abdominal tissues and organs. However, the process by which these larvae choose a specific organ or tissue to inhabit and how they cross the barrier between the liver and lungs to enter the bloodstream is still being studied. The most typical clinical sign is vague abdominal pain, which is caused by the cyst's gradual expansion. Considering that peritoneal cysts can result in peritonitis and anaphylactic shocks, this could result in a life-threatening diagnostic delay [2]. This case report mainly aims to identify hydatid cysts as they are asymptomatic for a long time.

## Case presentation

A 42-year-old man complained of gradual distension of the abdomen for six months when he arrived at the outpatient clinic. Our patient was a farmer. He had no complaints of abdominal pain, loss of appetite, yellowish discoloration of sclera, and lower limb edema. The patient did not report any fever, weight loss, or symptoms of night sweats. There was no history of headache, dizziness, or blurred vision. He had no history of diabetes, hypertension, bronchial asthma, or tuberculosis. The patient was a chronic alcoholic for 10 years, with an intake per day of 250 mL. His viral markers for hepatitis B, hepatitis C, and human immunodeficiency virus were all negative.

Upon physical examination, the patient's blood pressure was 110/70 mmHg, pulse rate was 102 bpm, and saturation at ambient room air was 98%. There were no symptoms of pedal edema, clubbing, icterus, cyanosis, or lymphadenopathy. The patient was discovered to have splenomegaly and hepatomegaly upon abdominal examination. Other systems were unremarkable. The table below lists every laboratory investigation (Table 1).

**Table 1:** Laboratory investigations of the patient cumm: cubic millimeter; dL: deciliter; fL: femtoliter; gm: gram; mg: milligram; mm: millimeter; mmol: millimole; pg: picogram; U/L: units per liter

Laboratory investigations	Value in the patient	Biological reference range
Hemoglobin	11.2	13-15 g/dL
Total Leukocyte Count	8220	4000-11,000/cumm
Platelet Count	327000	150,000-450,000/cumm
RBC Count	3.32	4.2-5.5 million/cumm
Hematocrit	36.3	35-52%
Mean Corpuscular Volume	79.3	79-100 fL
Mean Corpuscular Hemoglobin	28.9	27-34 pg
Mean Corpuscular Hemoglobin Concentration	32.9	31-36 gm/dL
Neutrophils	64	40-75%
Lymphocytes	22	20-45%
Eosinophils	5.4	1-6%
Monocytes	8.4	2-10%
Basophils	0.1	1%
Urea	14.5	9-20 mg/dL
Creatinine	1.1	0.6-1.2 mg/dL
Sodium	138	135-145 mmol/L
Potassium	4.4	3.5-5.1 mmol/L
Alkaline Phosphatase	67	38-126 unit/L
Alanine Transaminase	12.7	<50 U/L
Aspartate Transaminase	13.1	17-59 U/L
Total Protein	9.48	6.2-8.3 gm/dL
Albumin	2.1	3.4-5 gm/dL
Total Bilirubin	0.20	0.2-1.2 mg/dL
Globulin	7.47	2.3-3.5 gm/dL
Erythrocyte Sedimentation Rate	68	<15 mm/hr
Activated Partial Thromboplastin Clotting Time	30.6	29.5 control
Prothrombin Time	12.9	11.9 control
International Normalized Ratio	1.09	0.8-1.2

**Figure 1:**
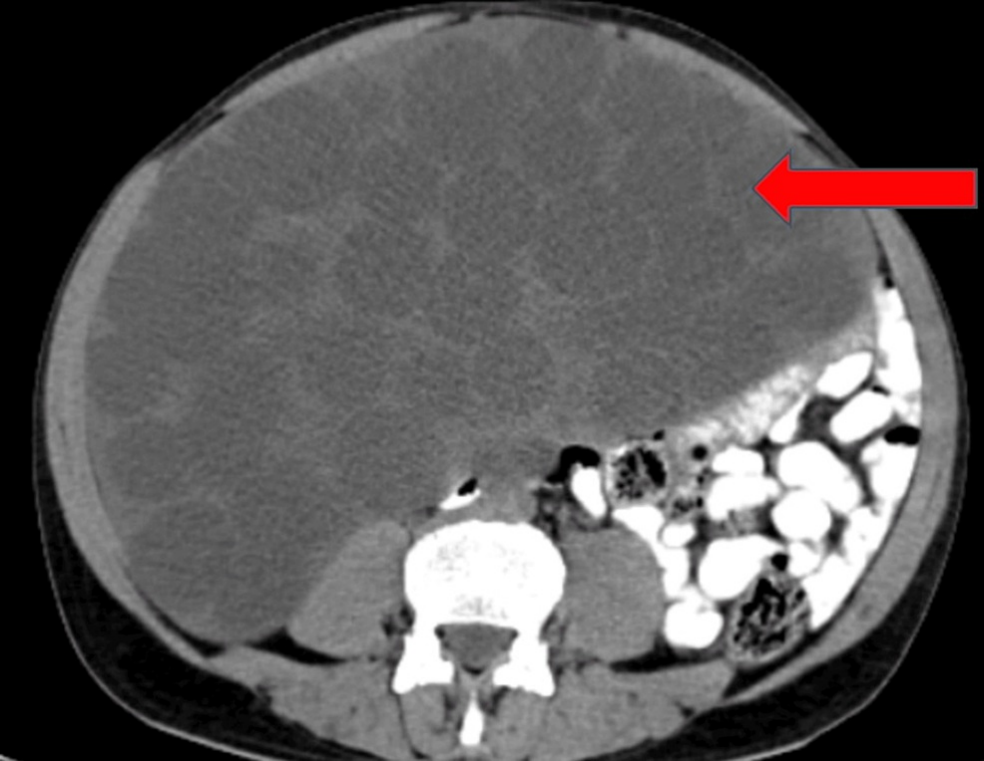
A CT of the abdomen showing multiple hydatid cysts in the peritoneum (red arrow)

**Figure 2:**
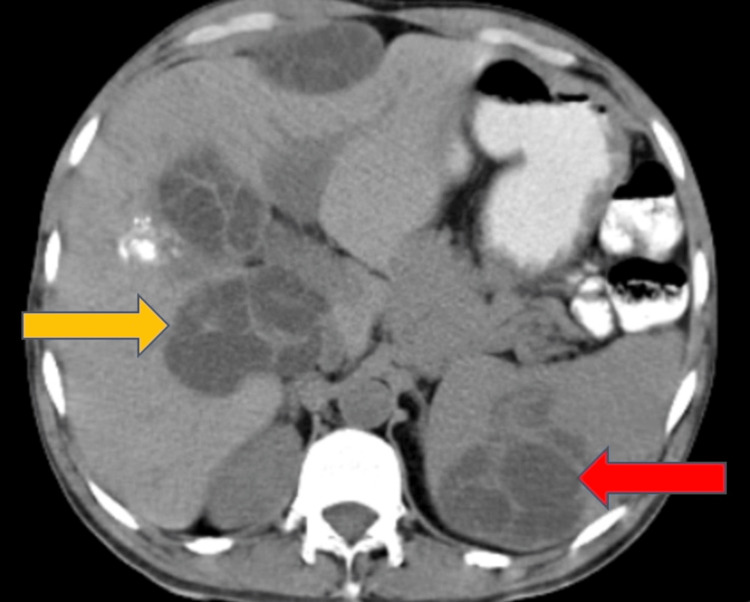
A CT of the abdomen showing hydatid cyst in the liver (yellow arrow) and the spleen (red arrow)

Multiple peritoneal hydatid cysts and hepatic and splenic hydatid cysts were visible on abdominal CT (Figures 1, 2).

The patient was prescribed oral albendazole 400 mg twice daily for 14 days and told that surgery would be necessary to remove the cysts, but the patient refused to have the cysts removed. As a result, the patient was given medical care and asked to follow up, but the patient was lost to follow-up.

## Discussion

The metacestode phase of Echinococcus tapeworms, belonging to the Taeniidae family, is the source of infection that causes hydatidosis. In communities where agriculture and animal husbandry are prevalent, hydatid cysts are frequently observed [3]. Hydatid disease remains a significant public health concern. Sheep, cattle, and deer are the intermediate hosts, whereas dogs and wolves are the primary carriers [3]. Upon ingestion, the eggs in the stomach shed their covering layer and release the developing embryos. Through the portal vein, the embryos travel from the intestinal mucosa to the liver. On rare occasions, some larvae may make it to the lungs, where they may then enter the bloodstream by slipping past the liver's and lungs' capillary filters. Hydatid cysts have the potential to develop in various organs throughout the body. However, they are predominantly found in the lungs and liver. The larvae rarely affect areas such as the spleen, kidney, pancreas, heart, ovaries, prostate, muscles, bones, and soft tissues [4]. Notably, peritoneal hydatidosis is a rare yet dangerous consequence of hydatidosis. In the rupture of a hydatid cyst of hepatic origin causes peritoneal echinococcosis. Traumatic rupture is primarily caused by an iatrogenic event that occurs during surgery or a diagnostic technique like transhepatic cholangiography or liver biopsy. It can also result from abdominal trauma or traffic accidents [5]. Complications of hydatid cysts are rare, and they may include spontaneous rupture of the cyst, which leads to anaphylaxis and secondary bacterial infection [6].

Diagnosing peritoneal hydatidosis requires the use of medical imaging. These days, many effective imaging techniques are available, and the resulting images are frequently very suggestive. For identifying and diagnosing hydatid disease in its abdominal sites, ultrasound is considered the first-line examination because of its reliability. It has been demonstrated that ultrasound helps identify one or more primary intraperitoneal hydatid localizations in cases of isolated peritoneal hydatidosis and reveals the primary hydatid cyst in patients of secondary peritoneal hydatidosis. Ultrasound makes it feasible to investigate the interaction between the hydatid cyst and inferior vena cava, suprahepatic veins, upper urinary tract, and portal bifurcation to look for potential compression [7]. CT has completely changed the detection of lesions and topographic diagnostics in abdominal hydatidosis as well as therapy. When compared to ultrasound, abdominal CT provides a simpler and far more accurate diagnosis of the hydatid cyst, mainly when it is in the peritoneum. Magnetic resonance imaging (MRI) makes it simple to diagnose rupture and track the progression of the hydatid cyst while receiving medical care [8]. Albendazole is the main antihelminthic drug given for the treatment [9]. The primary method of treating peritoneal hydatidosis remains to be surgery. The goal is to concurrently treat both the primary hydatid cysts and peritoneal cysts. As one can never be sure of having eliminated the hydatid cysts, it is coupled with medical care preoperatively for sterilizing the cysts and especially in postoperative scenarios to avoid recurrences, which are relatively frequent in peritoneal hydatidosis. Treatment can be medical, PAIR (puncture, aspiration, instillation, and reaspiration), or surgical. In surgery, it is necessary to remove all or a portion of the diseased organ. Albendazole used both before and after surgery lowers the recurrence rate [10]. On a histopathology examination, the hydatid cyst wall has an outer laminated membrane and an inner germinal layer. Protoscolices and brood capsules can be seen. The microscopic characteristics can differentiate it from other cysts that may affect the spleen such as benign cystadenoma [11].

## Conclusions

Hydatid cyst lesions can develop anywhere in the body because of E. granulosus. Therefore, unless otherwise demonstrated, cystic echinococcosis must be considered a differential diagnosis in individuals arriving with cystic enlargements anywhere on the body in endemic areas. Appropriate tests must be conducted to determine a precise diagnosis and treatment. Through meticulous surgical resection, complemented by adjunctive therapies such as albendazole, favorable outcomes can be achieved, mitigating the risk of recurrence and minimizing morbidity.

## References

[1] Romig T, Deplazes P, Jenkins D (2017). Chapter five - ecology and life cycle patterns of echinococcus species. Adv Parasitol.

[2] Conraths FJ, Deplazes P (2015). Echinococcus multilocularis: epidemiology, surveillance and state-of-the-art diagnostics from a veterinary public health perspective. Vet Parasitol.

[3] Bhutani N, Kajal P (2018). Hepatic echinococcosis: a review. Ann Med Surg (Lond).

[4] Dehkordi AB, Sanei B, Yousefi M, Sharafi SM, Safarnezhad F, Jafari R, Darani HY (2019). Albendazole and treatment of hydatid cyst: review of the literature. Infect Disord Drug Targets.

[5] Sachar S, Goyal S, Goyal S, Sangwan S (2014). Uncommon locations and presentations of hydatid cyst. Ann Med Health Sci Res.

[6] Alexiou K, Mitsos S, Fotopoulos A (2012). Complications of hydatid cysts of the liver: spiral computed tomography findings. Gastroenterology Res.

[7] Yilmaz M, Akbulut S, Kahraman A, Yilmaz S (2012). Liver hydatid cyst rupture into the peritoneal cavity after abdominal trauma: case report and literature review. Int Surg.

[8] Alshoabi SA, Alkalady AH, Almas KM (2023). Hydatid disease: a radiological pictorial review of a great neoplasms mimicker. Diagnostics (Basel).

[9] Ramos G, Orduña A, García-Yuste M (2001). Hydatid cyst of the lung: diagnosis and treatment. World J Surg.

[10] Abbasi B, Akhavan R, Ghamari Khameneh A (2021). Computed tomography and magnetic resonance imaging of hydatid disease: a pictorial review of uncommon imaging presentations. Heliyon.

[11] Hegazy RA, Hegazy AA, Alsayed SF, Ammar EE (2016). A rare case of splenic benign mucinous cystadenoma. Acad Anat Int.

